# Structural and functional divergence of the *Mpc1* genes in wheat and barley

**DOI:** 10.1186/s12862-019-1378-3

**Published:** 2019-02-26

**Authors:** Ksenia V. Strygina, Elena K. Khlestkina

**Affiliations:** 1 Institute of Cytology and Genetics, Siberian Branch of the Russian Academy of Sciences, Lavrentjeva ave. 10, Novosibirsk, 630090 Russia; 2 Novosibirsk State University, Pirogova str., 1, Novosibirsk, 630090 Russia; 3 N.I. Vavilov All-Russian Research Institute of Plant Genetic Resources (VIR), Bolshaya Morskaya Str., 42-44, St. Petersburg, 190000 Russia

**Keywords:** Anthocyanin biosynthesis, Flavonoid pigments, Gene duplication, Gene evolution, *Hordeum*, Myb, Near-isogenic lines, Transcription factor, *Triticum*

## Abstract

**Background:**

The members of the Triticeae tribe are characterised by the presence of orthologous and homoeologous gene copies regulating flavonoid biosynthesis. Among transcription factors constituting a regulatory MBW complex, the greatest contribution to the regulation of flavonoid biosynthetic pathway is invested by R2R3-Myb-type TFs. Differently expressed R2R3-Myb copies activate the synthesis of various classes of flavonoid compounds in different plant tissues. The aim of this research was the identification, comparison and analysis of full-length sequences of the duplicated R2R3-Myb *Mpc1* (*Myb protein c1*) gene copies in barley and wheat genomes.

**Results:**

The *Mpc1* genes were identified in homoeologous group 4 and 7 chromosomes: a total of 3 copies in barley (*Hordeum vulgare* L.) and 8 copies in bread wheat (*Triticum aestivum* L.) genomes. All *Mpc1* genes have a similar two-exon structure, and almost all of them are transcriptionally active. The calculation of the divergence time revealed that first duplication between 4 and 7 chromosomes of the common ancestor of the Triticeae tribe occurred about 35–46 million years ago (MYA); the last duplication arised about 16–19 MYA before the divergence *Triticum* and *Hordeum* genera The connection between gene expression and the appearance of anthocyanin pigmentation was found for three genes from homoeologous group 4 chromosomes: *TaMpc1-A2* (5AL) in wheat coleoptile, *HvMpc1-H2* (4HL) in barley lemma and aleurone layer, and *HvMpc1-H3* (4HL) in barley aleurone layer. *TaMpc1-D4* (4DL) from the wheat genome showed a strong level of expression regardless of the colour of coleoptile or pericarp. It is assumed, that this gene regulates the biosynthesis of uncoloured flavonoids in analysed tissues.

**Conclusions:**

The regulatory *R2R3-Myb* genes involved in anthocyanin synthesis were identified and characterised in Triticeae tribe species. Genes designated *HvMpc1-H2* and *HvMpc1-H3* appeared to be the main factors underlying intraspecific variation of *H. vulgare* by lemma and aleurone colour. *TaMpc1-A2* is the co-regulator of the *Mpc1–1* genes in bread wheat genome controlling anthocyanin synthesis in coleoptile.

**Electronic supplementary material:**

The online version of this article (10.1186/s12862-019-1378-3) contains supplementary material, which is available to authorized users.

## Background

Myb (myeloblastosis) family proteins belong to a large class of transcriptional regulators found in all eukaryotic organisms [1, 2]. The first identified proteins with Myb domains were oncogene of avian myeloblastosis virus (*v-myb*) and its cellular homolog (*c-myb*) [3, 4]. Myb proteins regulate diverse cellular processes, including cells growth and differentiation, stress response and biosynthesis of secondary metabolites [5–9]. All Myb factors share the presence of imperfect Myb repeats (R), which are involved both in binding DNA and in protein-protein interactions. R2R3-Myb containing two Myb repeats constitute the largest group of plant transcription factors [6, 10, 11]. They play an important role as expression regulators involved in the biosynthesis of flavonoid pigments.

**Fig. 1:**
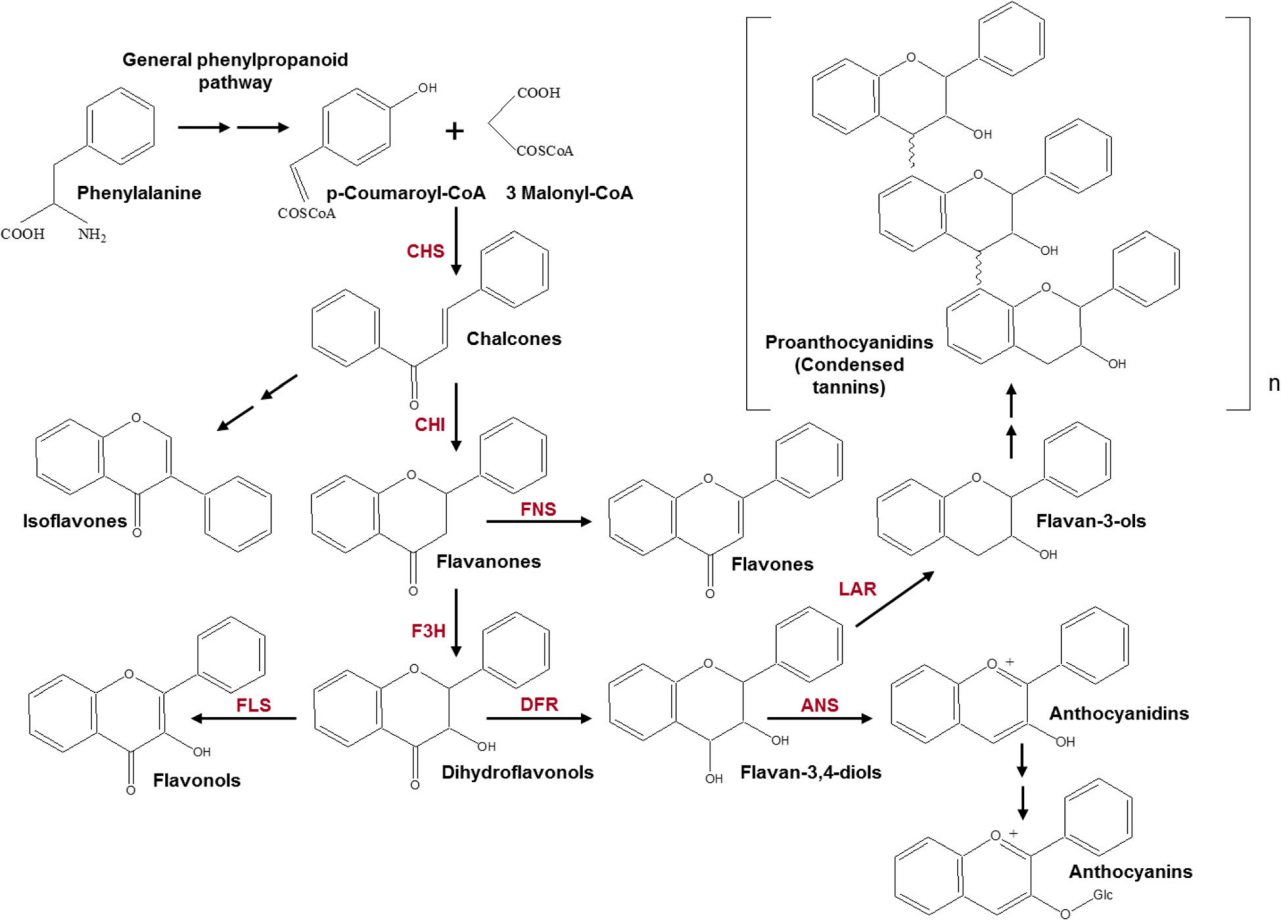
The scheme of flavonoid biosynthesis determining the colour of plants. The enzymes: ANS – anthocyanidin synthase; CHI – chalcone-flavanone isomerase; CHS – chalcone synthase; DFR – dihydroflavonol 4-reductase; F3H – flavanone 3-hydroxylase; FLS – flavonol synthase; FNS – flavone synthase; LAR – leucoanthocyanidin reductase

Flavonoids are plants secondary metabolites derived from the general phenylpropanoids pathway (Fig. 1) [12, 13]. The name of these components is associated with the Latin word “flavus” meaning “yellow”. In addition to yellow, flavonoid pigments colour plant tissues in pink, red, blue, purple and brown. The pathway of flavonoid biosynthesis generates several main groups of compounds: chalcones, flavones, isoflavones, flavanones, flavonols, flavan-3-ol, proanthocyanidins and anthocyanins (Fig. 1). Flavonoids have a broad spectrum of functions in plants such as growth regulation, protection against abiotic stress factors, pathogenic microbes and pests, and attraction of pollinators and seed distributors [12–16]. Mutations in *Myb* genes often lead to decrease of flavonoid biosynthesis genes expression and to colourless phenotype.

The *Myb*-type gene *Colourless1* (*c1*, or *Mpc1* – *Myb protein c1*) of maize located on chromosome 9 was the first identified gene encoding the transcription factor in plants [17]. Mutants of this gene are characterised by the absence of anthocyanin pigmentation in the aleurone layer of maize kernels. In 1999, Li et al. [18] have mapped a set of genes including *Mpc1* by hybridization of maize and rice probes on DNA of wheat nulli-tetrasomic lines. Maize *c1* homologs (named *Mpc1*) were detected in wheat chromosomes 4B, 4D, 5A and in homoeologous group 7 chromosomes [18]. Later it was revealed that the loci on homoeologous group 7 chromosomes (7AS, 7BS, 7DS) contain the *Myb*-type *TaMpc1* / *TaC1* genes, which have a pleiotropic effect – regulation of the colour of the coleoptile and the pericarp [19–21]. In barley, *c1* ortholog (*HvMpc1* / *HvAnt1*) regulating anthocyanin synthesis in the leaf sheath and the pericarp was identified on chromosome 7HS [20, 22, 23]. Shin et al. [24] have isolated CDS of the genes designated *TaPL1* (1 copy on 4BL, 3 copies on 4DL chromosomes) presumably involved in the regulation of anthocyanin biosynthesis in wheat coleoptile. It was shown that *TaPL1* is highly identical to *HvMpc1-H2* / *HvPL1* gene [24, 25].

The aim of the current study was the identification, phylogenetic analysis, as well as investigation of the structural and functional organisation of the *Mpc1* gene copies encoding transcription factors (TF) R2R3-Myb-type in bread wheat (*Triticum aestivum* L.), its relatives (diploid and tetraploid wheat of the *Triticum* genus, as well as diploid species of the *Aegilops* genus related to the genomes B and D of bread wheat) and barley (*Hordeum vulgare* L.).

## Results

### Identification of the *Mpc1* genes sequences of Triticeae tribe species

**Fig. 2:**
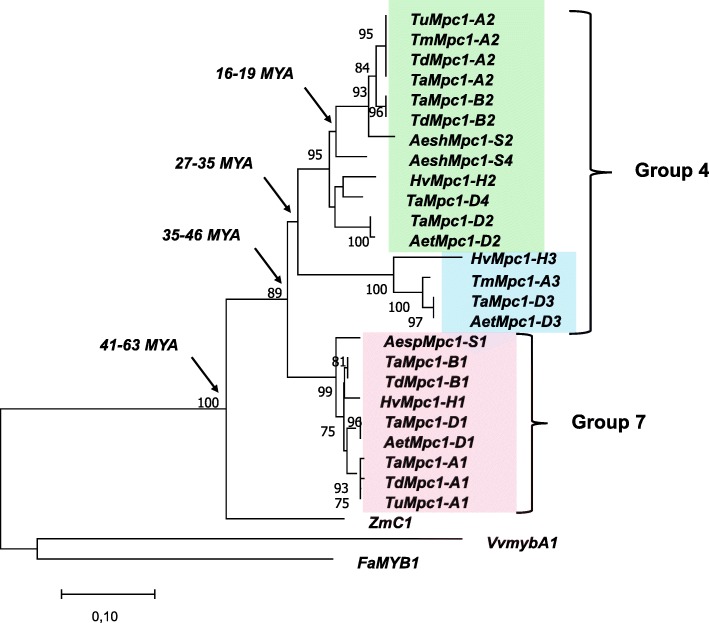
The phylogenetic similarity analysis of the *Mpc1* genes (CDS). The Neighbor-Joining method was used for construction of phylogenic tree in MEGA 6.06 with 1000 bootstrap replicates. *ZmC1* – NM_001112540, *VvmybA1* – AB097923, *FaMYB1* – AF401220. MYA – million years ago

Using the sequence of the *T. aestivum TaC1* gene and the *H. vulgare HvAnt1*, eight gene copies in the bread wheat genome and three copies in the barley genome were found by BLAST searching (Table 1, the information about the chromosomal localisation of various *Mpc1* genes was taken from IPK Barley BLAST Server for *Hordeum* contigs and International URGI database for *Triticum* and *Aegilops* contigs). One copy in barley 7HS chromosome and three copies in 7AS, 7BS and 7DS chromosomes of wheat were named *HvMpc1-H1* and *TaMpc1-A1*, *TaMpc1-B1*, *TaMpc1-D1* (Fig. 2) according to the rules for the designation of homoeologous genes [26]. The root “*Mpc1*” instead if “*C1*” or “*Ant1*” was used in the gene names according to the first description and designation (*Mpc1*) of the *C1* homologs in Triticeae tribe [18]). These genes have orthologs in di- and tetraploid species (Fig. 2, pink cluster).

**Table 1:** The regulatory *Mpc1* genes annotated in the current study. *Hordeum* genes were detected in IPK Barley BLAST Server, *Triticum* and *Aegilops* genes were detected in International URGI database

Gene	Previously designation	Organism	Chromosome	Contig
*HvMpc1-H1*	*HvAnt1*, *HvC1*	*Hordeum vulgare*	7HS	morex_contig_137164
*TuMpc1-A1*	*–*	*Triticum urartu*	7AS	TGAC_WGS_urartu_v1_contig_231166
*TmMpc1-A1*	*–*	*Triticum monococcum*	7AS	TGAC_WGS_monococcum_v1_contig_12604
*AespMpc1-S1*	*–*	*Aegilops speltoides*	7SS	TGAC_WGS_speltoides_v1_contig_38854
*AetMpc1-D1*	*–*	*Aegilops tauschii*	7DS	TGAC_WGS_tauschii_v1_contig_93382
*TdMpc1-A1*	*–*	*Triticum durum*	7AS	TGAC_WGS_durum_v1_contig_176466
*TdMpc1-B1*	*–*	*Triticum durum*	7BS	TGAC_WGS_durum_v1_contig_203087
*TaMpc1-A1*	*TaC1-A1*	*Triticum aestivum*	7AS	IWGSC_chr7AS_ab_k71_contigs_longerthan_200_4108742
*TaMpc1-B1*	*TaC1-B1*	*Triticum aestivum*	7BS	IWGSC_chr7BS_ab_k71_contigs_longerthan_200_3084267
*TaMpc1-D1*	*TaC1-D1*	*Triticum aestivum*	7DS	IWGSC_chr7DS_ab_k71_contigs_longerthan_200_2723852
*HvMpc1-H2*	*HvMpc2*, *HvPL1*	*Hordeum vulgare*	4HL	morex_contig_317820
*TuMpc1-A2*	*–*	*Triticum urartu*	4AL	TGAC_WGS_urartu_v1_contig_39917
*TmMpc1-A2*	*–*	*Triticum monococcum*	4AL	TGAC_WGS_monococcum_v1_contig_152763
*AeshMpc1-S2*	*–*	*Aegilops sharonensis*	4SL	TSL_WGS_sharonensis_v1_contig_100032
*AetMpc1-D2*	*–*	*Aegilops tauschii*	4DL	TGAC_WGS_tauschii_v1_contig_117718
*TdMpc1-A2*	*–*	*Triticum durum*	4AL	TGAC_WGS_strongfield_v1_contig_168597
*TdMpc1-B2*	*–*	*Triticum durum*	4BL	TGAC_WGS_durum_v1_contig_401905
*TaMpc1-A2*	*–*	*Triticum aestivum*	5AL	TGACv1_scaffold_374597_5AL
*TaMpc1-B2*	*TaPL1–4B1*	*Triticum aestivum*	4BL	IWGSC_chr4BL_ab_k71_contigs_longerthan_200_6843762
*TaMpc1-D2*	*TaPL1–4D1*	*Triticum aestivum*	4DL	IWGSC_chr4DL_V3_ab_k71_contigs_longerthan_200_14366448
*HvMpc1-H3*	*–*	*Hordeum vulgare*	4HL	morex_contig_1560519
*TmMpc1-A3*	*–*	*Triticum monococcum*	4AL	TGAC_WGS_monococcum_v1_contig_908028
*AetMpc1-D3*	*–*	*Aegilops tauschii*	4DL	TGAC_WGS_tauschii_v1_contig_1005499
*TaMpc1-D3*	*TaPL1–4D3*	*Triticum aestivum*	4DL	IWGSC_chr4DL_V3_ab_k71_contigs_longerthan_200_14406147
*AeshMpc1-S4*	*–*	*Aegilops sharonensis*	4SL	TSL_WGS_sharonensis_v1_contig_92163
*TaMpc1-D4*	*TaPL1–4D2*	*Triticum aestivum*	4DL	IWGSC_chr4DL_V3_ab_k71_contigs_longerthan_200_14366997

Five gene copies were found in the homoeologous group 4 chromosomes: one copy in 5AL (5A / 4A translocation region [27]), one copy in 4BL and three copies in 4DL (Table 1). Three of these genes represent the homoeologous set of *Mpc1–2* genes: *TaMpc1-A2* (5AL), *TaMpc1-B2* (4BL) and *TaMpc1-D2* (4DL) (Fig. 2, green cluster). This copy was preserved in related species, including barley – the gene *HvMpc1-H2*. Other copies named *Mpc1–3* and *Mpc1–4* were revealed in chromosomes 4 of the Triticeae tribe. We identified *TaMpc1-D3* gene of bread wheat and its orthologs in barley (*HvMpc1-H3*), *Triticum monococcum* and *Aegilops tauschii* genomes (Table 1, Fig. 2, blue cluster). The *Mpc1–4* genes were found only in *T. aestivum* (*TaMpc1-D4*) and *Aegilops sharonensis* genomes (Table 1, Fig. 2, green cluster). The level of identity between *Mpc1–1* and *Mpc1–2*, *Mpc1–3* and *Mpc1–4* nucleotide sequences is about 70%.

### Phylogenetic relationship of *Mpc1* genes

The *Mpc1–1* and *Mpc1–2* clusters contain orthologous genes of both polyploid and diploid species of the Triticeae tribe, including donors of A, B and D genomes of *T. aestivum* and barley *H. vulgare.* Within these clusters, it is possible to trace the fact that the orthologous genes belonging to one subgenome (A, B or D) fall into one phylogenetic group with the exception of the genes identified in the genomes of *Ae. sharonensis* and *Ae. speltoides* (Fig. 2). Most likely, these genes are separated from the orthologous genes represented in the *T. aestivum* and *T. durum* genomes due to the fact that *Ae. sharonensis* and *Ae. speltoides* are not direct donors of the B-genome of polyploid wheat species (*Ae. speltoides* is considered to be the closest to the ancestor of the B-genome among the existing cereals [28]. This dependence was not found in *Mpc1–3* cluster due to its smaller size (Fig. 2). Apparently, the *Mpc1–3* gene was most susceptible to pseudogenization in various members of the Triticeae tribe.

We assumed that the *Mpc1–1* and *Mpc1–2* genes are the result of the duplication of the *Mpc1* gene in the chromosomes 4 and 7 of the common diploid ancestor of the Triticeae tribe. The analysis of the genetic similarity of the *Mpc1* genes and the calculation of the divergence time revealed that this duplication occurred about 35–46 million years ago (MYA) (Fig. 2). The *Mpc1–3* gene copy occurred about 27–35 MYA due to segmented duplication in chromosome 4 of the common ancestor of the Triticeae tribe (Fig. 2). In addition, the *Mpc1–2* gene was again duplicated in some genomes at least in the genome D and S resulting in appearance of the *Mpc1–4* copy about 16–19 MYA before the divergence *Triticum* and *Hordeum* genera (approximately 9–11 MYA [29, 30]) (Fig. 2). In addition, we estimated the divergence time of *Zea* and *Hordeum* genomes. It occurred about 41–63 MYA, which is close to a known divergence time between these species – approximately 50–60 MYA [31].

### Structural organisation of the *Mpc1* genes

All identified genes have a site encoding the R2-R3 motif, which is characteristic for the *R2R3-Myb *subfamily. The exon-intronic structure for all the detected copies (*Mpc1–1*, *Mpc1–2*, *Mpc1–3*, *Mpc1–4*) is the same in all studied members of Triticeae tribe and consisted of two exons (Additional file 1). In the coding sequences of these genes only synonymous single nucleotide substitutions in all sequenced genotypes were detected.

Analysis of the promoters of these genes (~ 600 bp from the start codon) revealed that all the identified sequences have many Myc- and Myb-binding motifs and light-dependent elements required for the flavonoids biosynthesis (Additional file 2). Also, in the promoter of *HvMpc1-H3* some single nucleotide substitutions were revealed in BW, BA, PLP, OWB-Dom and OWB-Rec genotypes, as well as the 17 bp-insertion (CAGCAGAGCACTAGCTC) in the OWB-Rec genotype (Additional file 1).

### Functional organisation of wheat *Mpc1* genes

The expression of *Mpc1–1* genes was studied earlier [24]. Here we analysed the expression patterns of the *TaMpc1–2*, *TaMpc1–3*, *TaMpc1–4* genes using gene-specific primers developed. *TaMpc1-A2*, *TaMpc1-B2* and *TaMpc1-D2* showed transcriptional activity in the coleoptile, *TaMpc1-D4* gene was expressed both in the coleoptile and in the pericarp. All these genes were not transcribed in roots. In addition, the *TaMpc1-D3* gene expressed neither in coleoptile nor the pericarp.

**Fig. 3:**
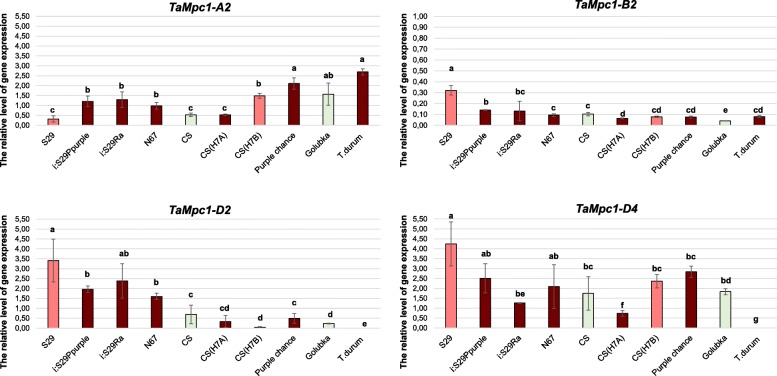
The expression of the *TaMpc1–2* and *TaMpc1–4* genes in wheat coleoptiles having different colouration (fifth day after germination). The colour of the column corresponds to the colour of wheat coleoptile. The data represent the means ± standard error of three biological replicates. Statistical significance was determined by one-way analysis of variance. Significant differences between means are indicated where letters above the bars differ (*p* ≤ 0.05)

Using quantitative RT-PCR we determined the relative level of genes expression in wheat cultivars and lines differing by anthocyanin pigmentation (Fig. 3, Table 2). It was shown that the level of expression of the *TaMpc1-A2* gene correlates with the anthocyanin colouration of the coleoptile for almost all analysed wheat varieties. The *TaMpc1-B2* gene shows a weak level of expression in the coleoptile of all analysed genotypes regardless of the pigmentation. The *TaMpc1-D4* gene expression was also independent on coleoptile colour, but overall it was much stronger compared to that of *TaMpc1-B2* (the 8th genotype, *T.durum*, is not considered in case of interpretation of the D-genomic gene’s expression, since it lacks the D-genome). The *TaMpc1-D2* gene expression levels varied significantly among genotypes, however this variation was not related with the coleoptile colour.

**Table 2:** Genetic stocks of wheat transcription that were used to characterise the *Mpc1* genes. NIL – near-isogenic line, SCSL – single chromosome substitution line

Cultivar/line designation	Genome	Description	Anthocyanin pigmentation of coleoptile	Anthocyanin pigmentation of pericarp
i:S29 *pp-A1pp-D1pp3* (S29/YP 140)	BBAADD	Wheat NIL developed on S29, donor YP 140	uncoloured	uncoloured
i:S29*Pp-A1pp-D1pp3* (Saratovskaya 29, S29)	BBAADD	Russian spring wheat	light red colour	uncoloured
i:S29*Pp-A1Pp-D1Pp3*^P^ (i:S29Ppurple)	BBAADD	Wheat NIL developed on S29, donor Purple	dark red colour	dark purple colour
i:S29*Pp-A1Pp-D1pp3*^P^	BBAADD	Wheat NIL developed on S29, donor Purple	dark red colour	uncoloured
i:S29*Pp-A1pp-D1Pp3*^P^	BBAADD	Wheat NIL developed on S29, donor Purple	light red colour	light purple colour
i:S29*Pp-A1Pp-D1Pp3*^PF^ (i:S29Ppurple)	BBAADD	Wheat NIL developed on S29, donor Purple Feed	dark red colour	dark purple colour
i:S29*Pp-A1Pp-D1pp3*^PF^	BBAADD	Wheat NIL developed on S29, donor Purple Feed	dark red colour	uncoloured
i:S29*Pp-A1pp-D1Pp3*^PF^	BBAADD	Wheat NIL developed on S29, donor Purple Feed	light red colour	light purple colour
i:S29Ra	BBAADD	Wheat NIL developed on S29, donor Ulyanovka	dark red colour	uncoloured
Novosibirskaya 67 (N67)	BBAADD	Russian spring wheat	dark red colour	uncoloured
Chinese Spring (CS)	BBAADD	Chinese spring wheat	uncoloured	uncoloured
Chinese Spring (Hope 7A) (CS(H7A))	BBAADD	Wheat SCSL developed on CS, donor Hope	dark red colour	uncoloured
Chinese Spring (Hope 7B) (CS(H7B))	BBAADD	Wheat SCSL developed on CS, donor Hope	light red colour	uncoloured
Purple chance	BBAADD	Russian spring wheat	dark red colour	dark purple colour
Golubka	BBAADD	Russian spring wheat	uncoloured	uncoloured
TRI 15744	BBAA	Triticum durum wheat	dark red colour	dark purple colour

**Fig. 4:**
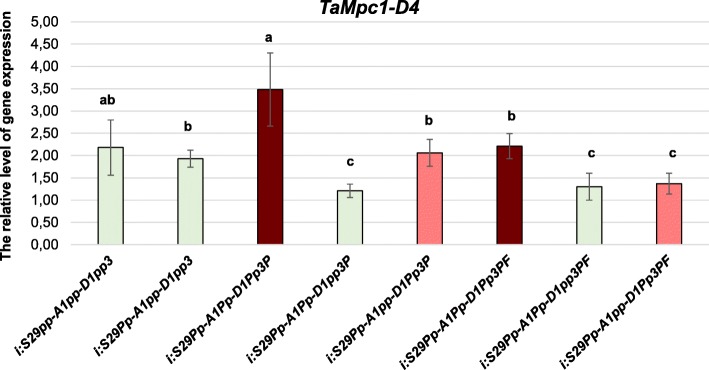
The expression of *TaMpc1-D4* in wheat pericarps having different colouration. The colour of the column corresponds to the colour of wheat coleoptile. The data represent the means ± standard error of three biological replicates. Statistical significance was determined by one-way analysis of variance. Significant differences between means are indicated where letters above the bars differ (p ≤ 0.05)

Also, we analysed the level of *TaMpc1-D4* expression in pericarps of wheat lines, differing in the presence of dominant *Pp-A1*, *Pp-D1* and *Pp3* genes (*TaMpc1-A1*, *TaMpc1-D1* and *TaMyc-A1*, respectively) controlling the anthocyanin biosynthesis in this tissue (Table 2). *TaMpc1-D4* is expressed intensively in the pericarp of all genotypes possessing both pigmented and uncoloured pericarp (Fig. 4).

### Functional organisation of barley *Mpc1 *genes

**Fig. 5:**
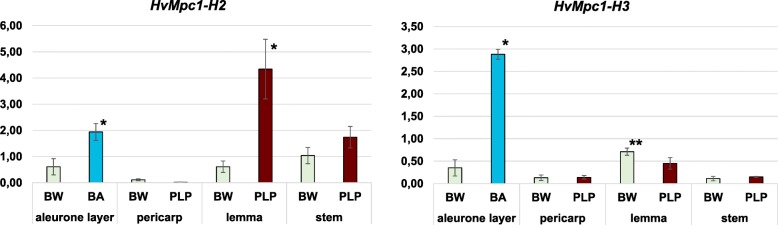
The expression of *HvMpc1-H2* and *HvMpc1-H3* in barley aleurone layer, pericarp, lemma and stems of Bowman NIL contrasting in anthocyanin pigmentation. The data are presented as mean value ± standard error. Statistical significance was determined by *U*-test. *differences are statistically significant between Bowman and NILs at *p* ≤ 0.005. **differences are statistically significant between Bowman and NILs at *p* ≤ 0.05

The *Mpc1-H2* gene expression was studied earlier [25]. Here we investigated the relative levels of expression of the *HvMpc1-H2* and *HvMpc1-H3* genes in the aleurone layer, pericarp, lemma and stems of Bowman near isogenic lines (NILs) contrasting in anthocyanin pigmentation (Table 3). Both genes had very low activity in the pericarp. *HvMpc1-H3* had in addition low activity in stems. *HvMpc1-H2* was characterised by high transcriptional activity in the aleurone layer, lemma and stems, and its expression level correlated with the colour of the aleurone layer and lemma (Fig. 5). The highest expression level was detected in lemma in coloured NIL (PLP), which was 7 times higher than in uncoloured one (BW). *HvMpc1-H3* has a high level of expression in the aleurone layer, and in the coloured aleurone (BA) the expression of this gene was ~ 8 times higher than in the uncoloured sample (Fig. 5). Also, this gene is expressed in the lemma of the analysed NILs. Interestingly, the expression of *HvMpc1-H3* was 1.5 times higher in the uncoloured BW than in PLP.

**Table 3:** Bowman near-isogenic lines (NILs) that were used in current research and their phenotypic characteristics. NGB – Nordic GenBank

NGB ID	Line designation	Anthocyanin pigmentation of analysed tissue			
		aleurone layer	pericarp	lemma	stem
NGB22812	BW (Bowman)	uncoloured	uncoloured	uncoloured	uncoloured
NGB20651	BA (Blue aleurone)	blue	uncoloured	uncoloured	purple
NGB22213	PLP (Purple lemma and pericarp)	uncoloured	purple	uncoloured	purple
–	OWB-Dom	blue	uncoloured	uncoloured	uncoloured
–	OWB-Rec	uncoloured	uncoloured	uncoloured	uncoloured

## Discussion

In addition to the chlorophyll colouration, plants organs could be coloured with polyphenolic flavonoid compounds. The biosynthesis of flavonoids is one of the most fully characterised plant metabolic pathways (Fig. 1). In the Triticeae tribe, the main structural genes encoding the enzymes of the flavonoid biosynthesis pathway are identified and localised [23, 32–34]. However, regulatory genes encoding Myb, bHLH/Myc and WD40 transcription factors (forming the MBW complex) are poorly studied. These factors provide tissue-specific expression of structural genes, and, consequently, tissue-specific accumulation of flavonoid pigments. In this study, we carried out the identification and characterization of R2R3-Myb-coding genes, potentially involved in the biosynthesis of flavonoids in Triticeae tribe.

To date, the genes encoding TF R2R3-Myb-type associated with anthocyanin biosynthesis and localised in homoeologous group 7 chromosomes (*TaMpc1–1* in the wheat chromosomes 7A, 7B and 7D and *HvMpc1-H1* in the barley chromosome 7H [19]). However, the data obtained earlier with Southern blot analysis on wheat nulli-tetrasomic lines [18] indicated the presence of homologs of the *Mpc1* genes in homoeologous group 4 chromosomes. Using the sequence of the *Mpc1–1* genes, 5 additional gene copies in 5A, 4B and 4D chromosomes of bread wheat and 2 additional copies in 4H chromosome of barley were identified. We demonstrated these genes to be divided into three main clusters, with *Mpc1–3* cluster being the smallest (Fig. 2). Apparently, *Mpc1–3* genes were the most susceptible to pseudogenization and to elimination from the genomes in Triticeae tribe.

It is known that the *Mpc1–1* genes are specific regulators of anthocyanin pigments biosynthesis [23, 32]. We determined the expression of previously unstudied *Mpc1–2*, *Mpc1–3* and *Mpc1–4* genes. It was shown that almost all *Mpc1* genes are transcriptionally active, and they are characterised by a tissue-specific expression pattern. The fact that none of the *Mpc1* genes of wheat is expressed in the roots indicates that probably there are other R2R3-Myb encoding genes in *T. aestivum* genome with a lower homology level providing the flavonoids biosynthesis in the roots.

An interesting fact was noted for *TaMpc1-A2* – its expression is associated with the colouration of coleoptile of bread wheat (Fig. 3). Potentially the transcript of this gene was previously detected and cloned from the wheat cultivar Iksan370, which preferentially expresses anthocyanins in coleoptiles [24], since none of the other *Mpc1–2*, *Mpc1–3* and *Mpc1–4* gene has demonstrated a correlation with the colouration of this tissue. It is known that the colour of coleoptile (as well as the colour of pericarp) is provided by the presence of dominant *Mpc1–1* genes mapped to homoeologous group 7 chromosomes [19–21]. *TaMpc1-A2* may be involved in the synthesis of uncoloured anthocyanin precursors in wheat coleoptile as a co-regulator of the *Mpc1–1* genes. *TaMpc1-D4* could be another regulator of uncoloured flavonoid compounds biosynthesis both in coleoptile and in pericarp (Figs. 3, 4).

Barley pigmentation of stems and pericarp is known to be associated with the dominant *HvMpc1-H1* [19, 21]. Probably, the expression of this gene causes tissue-specific suppression of *HvMpc1-H2* and *HvMpc1-H3* genes in the pericarp and suppression of *HvMpc1-H3* in the stems (Fig. 5). *HvMpc1-H2* is probably the main regulator of the appearance of anthocyanin pigmentation in the barley lemma. It is likely that the overexpression of *HvMpc1-H2* in the lemma suppresses the expression of *HvMpc1-H3* in the lemma of coloured PLP line (Fig. 5).

The blue colour of the aleurone layer depends on five complementary *Blx* (*Blue aleurone xenia*) genes. Three of these genes (*Blx1*, *Blx3*, *Blx4*) are closely linked to each other and were mapped to chromosome 4HL [35]. It was previously revealed that *Blx3* and *Blx4* appear to be bHLH-coding gene *HvMyc2* and Cytochrome P450-coding *HvF3’5’H* [25]. *HvMpc1-H3* is apparently the co-regulator of anthocyanin pigments accumulation in the barley aleurone layer (Fig. 5). In addition, the presence of a mutation in genotype OWB-Rec (insertion of 17 bp) was identified in the promoter of *HvMpc1-H3* (Additional file 1). If this gene is the missing R2R3-Myb component of the MBW complex initiating anthocyanins synthesis in the aleurone layer, then this mutation in *HvMpc1-H3* with previously identified mutation in *HvMyc2* in OWB-Rec genotype could be a reason of a digenic inheritance in blue colouration of the aleurone layer in OWB population [25]. Potentially, *HvMpc1-H3* is the third *Blx* gene (*Blx1*) from the 4HL chromosome.

## Conclusions

R2R3-Myb-coding genes involved in flavonoid synthesis in Triticeae tribe were identified and characterised, from which *TaMpc1-A2* is the co-regulator of the *Mpc1–1* genes in bread wheat genome controlling anthocyanin synthesis in coleoptile. *HvMpc1-H2* and *HvMpc1-H3* appeared to be the main factors underlying variation of barley lemma and aleurone colour, respectively.

## Methods

### Gene identification and in silico data analysis

The search of homologous sequences of *Mpc1* gene was made in databases for wheat (International URGI database, https://urgi.versailles.inra.fr) and barley (IPK Barley BLAST Server, http://webblast.ipkgatersleben.de/barley_ibsc/) sequences using BLASTN. Barley gene *HvMpc1-H1* (GenBank: KP265977) and wheat genes *TaMpc1–1* (GenBank: AB983540, AB983541, AB983542) were used to identify *Mpc1* sequences in barley and wheat genome, respectively. The exon-intronic structure of the genes was predicted with FGENESH+ software (http://www.softberry.com/berry.phtml?topic=fgenes_plus&group=programs&subgroup=gfs) using polypeptide sequence of homologous gene *HvMpc1-H1.* The sequences were aligned using Multalin (http://multalin.toulouse.inra.fr/multalin/). The construction of the Neighbour-joining tree was performed using MEGA v5.1 software (http://www.megasoftware.net) with 1000 bootstrap replicates to assess the branch support. Promoters of the genes were analysed with New PLACE database (https://sogo.dna.affrc.go.jp/cgi-bin/sogo.cgi?lang=en&pj=640&action=page&page=newplace). The *Ks* parameter (the frequency of synonymous substitutions) was estimated using modified Nei–Gojobori method [36]. The rate of nucleotide substitution accumulation (*k* = 3.2 × 10^− 9^) was calculated with the formula *Ks*/2 *T* according to [37] using 10 MYA as the average time of barley and wheat divergence*.* The average *Ks* value was calculated using *Ks*_(*HvMpc1-H1*/*TaMpc1-A1*)_ = 0.089, *Ks*_(*HvMpc1-H1*/*TaMpc1-B1*)_ = 0.044 and *Ks*_(*HvMpc1-H1*/*TaMpc1-D1*)_ = 0.059. The divergence time were calculated for the remaining genes according to the formula *T* = *Ks*/2 *k*.

### Plant materials

Plant material included three Bowman’s near-isogenic lines (NILs) and two parental lines (OWB-Dom and OWB-Rec) of barley *H. vulgare* (HH), one tetraploid line of the *T. durum* (BBAA) and sixteen lines and cultivars of the hexaploid wheat *T. aestivum* (BBAADD) (Tables 2, 3). The three NILs, *T. durum* line and sixteen *T. aestivum* cultivars and lines were exploited for gene expression analysis. CS, S29, OWB-Dom, OWB-Rec, BW and BA were also used for sequencing. The experiments were conducted in three replicates for each genotype. The plants for DNA extraction and RNA extraction from stems, aleurone layers, pericarps and lemmas were grown in ICG Greenhouse Core Facilities (Novosibirsk, Russia) under а 12 h of light per day at 20–25 °C. Seeds for RNA extraction were germinated on moist filter paper in the climatic chamber “Rubarth Apparate” (RUMED GmbH, Laatzen, Germany) under a 12 h photoperiod at 20 °C.

### Extraction of DNA and RNA, reverse transcription

Total genomic DNA was extracted from fresh leaves following [38]. Wheat and barley pericarps and barley aleurones were cut out with a scalpel from grains at early dough stage maturity for RNA extraction. RNAs from wheat coleoptile samples (fifth day after germination), barley lemmas, stems (collected at the end of flowering) and pericarps were extracted using a ZR Plant RNA MiniPrep™ (Zymo Research, USA), RNAs from aleurone layers were extracted using a RNeasy Mini Kit (QIAGEN, Germany) according to the manufacturer’s instructions. All isolated RNAs were treated with RNase-free DNase set (QIAGEN, Germany). To obtain single-stranded cDNA samples total RNA was converted in a 20-μL reaction mixture from a template consisting of 0.4 μg of total RNA using a RevertAid First Strand cDNA Synthesis Kit (Thermo Fisher Scientific Inc., USA).

### Primer design, PCR, qRT-PCR and sequencing

Gene-specific primer pairs were constructed using OLIGO 7 software based on sequences found in IPK Barley BLAST Server and International URGI database (Table 4). PCR was performed in a 20-μL reaction mixture containing 75 ng of gDNA or cDNA, 0.2 mM of each dNTP, 1.5 mM MgCl_2_, 67 mM Tris-HCl pH 8.8, 0.01% Tween 20, 18 mM (NH_4_)_2_SO_4_, 1 ng of each primer and one unit of Taq DNA polymerase (Medigen Ltd., Russia) with the following profile: 1 cycle at 94 °C for 2 min; 35 cycles at 94 °C for 1 min, 50–60 °C for 1 min, 72 °C for 2 min; 1 cycle at 72 °C for 5 min. The qRT-PCR was based on a SYNTOL SYBR Green I kit (Syntol, Moscow, Russia) with the primers from Table 4. The reference sequence was ubiquitin, primers were suggested in [39]. The amplifications were performed in an ABI Prism 7000 Sequence Detection System (Applied Biosystems, http://www.lifetechnologies.com). Each sample was run in three technical replications. PCR products were separated in 1% agarose gel. The amplified fragments were purified from an agarose gel using a DNA Clean kit (BioSilica, Novosibirsk, Russia).

**Table 4:** Gene-specific primers used for amplification and sequencing *Mpc1* gene sequences of barley and wheat

Gene	Forward primer (5′ → 3′)	Reverse primer (5′ → 3′)	Annealing temperature. (°C)	Purpose	PCR product length (bp) DNA/cDNA
*HvMpc1-H2*	GTAACAGGTGGTCGCTCATT	TTGGAGGAGACGGAGCTG	60	qPCR, gene sequencing	167/167
*HvMpc1-H3*	GAAGGCAGATGGAACGAAGT	GATGATGGACCACCTGTTG	60	qPCR, gene sequencing	238/177
	ATGAGGAAGGAAGGAGTGAAGA	TTATAGCGGCATGTCCACAGAG	55	gene sequencing	784/723
	GGACCGGGACTAATAGGATTTC	GTTTCGTCCTCCTTGCTAGTC	55	promoter sequencing	692/−
	GGCCTAACGAGCTGAAGTATT	GTTTCGTCCTCCTTGCTAGTC	55	promoter sequencing	319/−
*TaMpc1-A2*	CCGAACAGACAACGAAATCAAG	CCACCCTGGTGGCAGCT	60	qPCR, gene sequencing	83/83
	ATGAGGAGGGCGTGCAGT	TTAATCCGCCATGTGCAGGGA	55	gene sequencing	853/729
*TaMpc1-B2*	CCGAACAGACAACGAAATCAAG	CCGCAGTTAGAGGAAAGCCAT	60	qPCR, gene sequencing	162/162
	ATGGGGATGAGGACGTGCAG	TTAATACGCCATCTGCAGGGACT	55	gene sequencing	843/738
*TaMpc1-D2*	CCGAACAGACAACGAAATCAAG	GCGCCCACACCGCGAT	60	qPCR, gene sequencing	218/218
	ATGGGGAGGAGGGCGTGC	TTAATCTGCCATCTGCAGGGAGT	58	gene sequencing	847/732
*TaMpc1-D3*	CCGAACAGACAACGAAATCAAG	CAGGTCCAGAGCTAGACAGA	60	qPCR, gene sequencing	161/−
	ATGGCGACGGAAGGGGTGAAGA	TTAACCCTCTCACTTCGTGCATCC	58	gene sequencing	742/−
*TaMpc1-D4*	CCGAACAGACAACGAAATCAAG	GCCCCGGAGTTGGAGGA	60	qPCR, gene sequencing	132/132
	ATGGCGACGGAAGGGGTGAAGA	TTAACCCTCTCACTTCGTGCATCC	60	gene sequencing	791/675

## Additional files

Additional file 1: A. The exon-intronic structure for *Mpc1* genes of Triticeae tribe. B. The multiple sequence alignment of promotor sequences of *HvMpc1-H3*. The alignment was performed using MultAlin program. Red is high consensus colour, blue is low consensus colour, black is neutral colour. (PDF 71 kb)

Additional file 2: Putative *cis*-acting regulatory elements identified in the *Mpc1* promoters. Promoter analysis was performed using New PLACE database. “+” – coding strand, “–” – template strand. (PDF 326 kb)

## References

[1] Ambawat S, Sharma P, Yadav NR, Yadav RC (2013). MYB transcription factor genes as regulators for plant responses: an overview. Physiol Mol Biol Plants.

[2] Hong JC. General aspects of plant transcription factor families. Plant Transcription Factors. 2015:35–56. 10.1016/B978-0-12-800854-6.00003-8.

[3] Klempnauer KH, Gonda TJ, Bishop JM (1982). Nucleotide sequence of the retroviral leukemia gene v–myb and its cellular progenitor c–myb: the architecture of a transduced oncogene. Cell.

[4] Lipsick JS (1996). One billion years of Myb. Oncogene.

[5] Martin C, Paz-Ares J (1997). MYB transcription factors in plants. Trends Genet.

[6] Feller A, Machemer K, Braun EL, Grotewold E (2011). Evolutionary and comparative analysis of MYB and bHLH plant transcription factors. Plant J.

[7] Xu W, Dubos C, Lepiniec L (2015). Transcriptional control of flavonoid biosynthesis by MYB–bHLH–WDR complexes. Trends Plant Sci.

[8] Baldoni E, Genga A, Cominelli E (2015). Plant MYB transcription factors: their role in drought response mechanisms. Int J Mol Sci.

[9] Liu J, Osbourn A, Ma P (2015). MYB transcription factors as regulators of phenylpropanoid metabolism in plants. Mol Plant.

[10] Jin H, Martin C (1999). Multifunctionality and diversity within the plant MYB-gene family. Plant Mol Biol.

[11] Stracke R, Werber M, Weisshaar B (2001). The R2R3-MYB gene family in Arabidopsis thaliana. Curr Opin Plant Biol.

[12] Grotewold E (2006). The genetics and biochemistry of floral pigments. Annu Rev Plant Biol.

[13] Grotewold E (2006). The science of flavonoids.

[14] Khlestkina E (2013). The adaptive role of flavonoids: emphasis on cereals. Cereal Res Commun.

[15] Pourcel L, Routaboul J-M, Cheynier V, Lepiniec L, Debeaujon I (2007). Flavonoid oxidation in plants: from biochemical properties to physiological functions. Trends Plant Sci.

[16] Landi M, Tattini M, Gould KS. Multiple functional roles of anthocyanins in plant-environment interactions. Environ Exp Bot 2015;199:4–17. https://doi. org/10.1016/j.envexpbot.2015.05.012.

[17] Paz-Ares J, Ghosal D, Wienand U, Peterson PA, Saedler H (1987). The regulatory c1 locus of Zea mays encodes a protein with homology to myb proto–oncogene products and with structural similarities to transcriptional activators. EMBO J.

[18] Li WL, Faris JD, Chittoor JM, Leach JE, Hulbert SH, Liu DJ, Chen PD, Gill BS (1999). Genomic mapping of defense response genes in wheat. Theor Appl Genet.

[19] Himi E, Taketa S (2015). Isolation of candidate genes for the barley Ant1 and wheat Rc genes controlling anthocyanin pigmentation in different vegetative tissues. Mol Gen Genomics.

[20] Jiang W, Liu T, Nan W, Jeewani DC, Niu Y, Li C, Wang Y, Shi X, Wang C, Wang J, Li Y, Gao X, Wang Z (2018). Two transcription factors TaPpm1 and TaPpb1 co-regulate anthocyanin biosynthesis in purple pericarps of wheat. J Exp Bot.

[21] Gordeeva EI, Glagoleva AY, Kukoeva TV, Khlestkina EK, Shoeva OY. Purple-grained barley (Hordeum vulgare L.): marker-assisted development of NILs for investigating peculiarities of the anthocyanin biosynthesis regulatory network. BMC Plant Biol. 10.1186/s12870-019-1638-9 PMC6393963 30813902

[22] Shoeva OY, Kukoeva TV, Börner A, Khlestkina EK (2015). Barley Ant1 is a homolog of maize C1 and its product is part of the regulatory machinery governing anthocyanin synthesis in the leaf sheath. Plant Breed.

[23] Shoeva OY, Strygina KV, Khlestkina EK. Genes determining the synthesis of flavonoid and melanin pigments in barley. Vavilovskii Zhurnal Genetiki i Selektsii (Vavilov J Genet Breed). 2018;22(3):333–342. doi:10.18699/VJ18.369.

[24] Shin DH, Choi MG, Kang CS, Park CS, Choi SB, Park YI (2016). A wheat R2R3-MYB protein PURPLE PLANT1 (TaPL1) functions as a positive regulator of anthocyanin biosynthesis. Biochem Biophys Res Commun.

[25] Strygina KV, Börner A, Khlestkina EK (2017). Identification and characterization of regulatory network components for anthocyanin synthesis in barley aleurone. BMC Plant Biol.

[26] McIntosh RA, Dubcovsky J, Rogers JW, Morris CF, Appels R, Xia X. Catalogue of gene symbols for wheat: 2013-14 supplement. Annual wheat newsletter 2014;58.

[27] Devos KM, Dubcovsky J, Dvořák J, Chinoy CN, Gale MD (1995). Structural evolution of wheat chromosomes 4A, 5A, and 7B and its impact on recombination. Theor Appl Genet.

[28] Gill BS, Kimber G (1974). Giemsa C-banding and the evolution of wheat. PNAS.

[29] Cheng J, Khan MA, Qiu WM, Li J, Zhou H, Zhang Q, Gou W, Zhu T, Peng J, Sun F, Li S, Korban SS, Han Y (2012). Diversification of genes encoding granule-bound starch synthase in monocots and dicots is marked by multiple genome-wide duplication events. PLoS One.

[30] Subburaj S, Cao S, Xia X, He Z (2016). Phylogenetic analysis, lineage-specific expansion and functional divergence of seed dormancy 4-like genes in plants. PLoS One.

[31] Salse J, Abrouk M, Bolot S, Guilhot N, Courcelle E, Faraut T, Waugh R, Close TJ, Messing J, Feuillet C. Reconstruction of monocotelydoneous proto-chromosomes reveals faster evolution in plants than in animals. PNAS 2009;pnas-0902350106. doi:10.1073/pnas.0902350106. 10.1073/pnas.0902350106 PMC2736418 19706486

[32] Khlestkina EK, Shoeva OY, Gordeeva EI. Flavonoid biosynthesis genes in wheat. Russ J Genet Appl Res. 2015;5(3):268–78. 10.1134/S2079059715030077.

[33] Jende-Strid B (1993). Genetic control of flavonoid biosynthesis in barley. Hereditas.

[34] Adzhieva VF, Babak OG, Shoeva OY, Kilchevsky AV, Khlestkina EK. Molecular-genetic mechanisms underlying fruit and seed colouration in plants. Vavilovskii Zhurnal Genetiki i Selektsii (Vavilov J Genet Breed). 2015;19(5):561–573. doi:10.18699/VJ15.073.

[35] Finch RA, Simpson E (1978). New colours and complementary colour genes in barley. Z Pflanzenzücht.

[36] Nei M, Gojobori T (1986). Simple methods for estimating the numbers of synonymous and nonsynonymous nucleotide substitutions. Mol Biol Evol.

[37] Strygina KV, Khlestkina EK (2017). MYC gene family in cereals: transformations during evolution of hexaploid bread wheat and its relatives. Mol Biol.

[38] Plaschke J, Ganal MW, Röder MS (1995). Detection of genetic diversity in closely related bread wheat using microsatellite markers. Theor Appl Genet.

[39] Himi E, Noda K (2005). Red grain colour gene (R) of wheat is a Myb-type transcription factor. Euphytica.

