# Supplementary material for: Structural and functional divergence of the Mpc1 genes in wheat and barley
Source: BMC Evol Biol. 2019 Feb 26;19(Suppl 1):45. doi: 10.1186/s12862-019-1378-3 (PMC6391766; doi:10.1186/s12862-019-1378-3)
Supplement: Supplementary file 1 — A. The exon-intronic structure for Mpc1 genes of Triticeae tribe. B. The multiple sequence alignment of promotor sequences of HvMpc1-H3. The alignment was performed using MultAlin program. Red is high consensus colour, blue is low consensus colour, black is neutral colour. (PDF 71 kb) [file 12862_2019_1378_MOESM1_ESM.pdf]

Additional file 1.

A. The exon-intronic structure for *Mpc1* genes of Triticeae tribe.

B. The multiple sequence alignment of promotor sequences of *HvMpc1-H3*. The alignment was performed using MultAlin program. Red is high consensus colour, blue is low consensus colour, black is neutral colour.

A

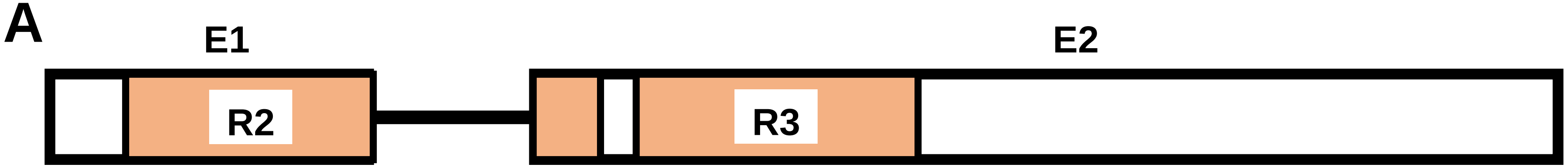

B

```
                 1                                                                                                                          130
HvMpc1-H3-BW     ACTGCACGGA CCTGCTGCTA TCGAGATAGG ACGAGGAGGT TAGCCACGAG CGTATCTGAG ACTGGGAGAC GAACCACGCG TAGCAGCAGA GCACTAGCTC ---------- -------ATG GCCCGCGCAT
HvMpc1-H3-BA     ACTGCACGGA CCTGCTGCTA TCGAGATAGG ACGAGGAGGT TAGCCACGAG GGTATCTGAG ACTGGGAGAC GAACCACGCG TAGCAGCAGA GCACTAGCTC ---------- -------ATG GCCCGCGCAT
HvMpc1-H3-PLP    ACTGCACGGA CCTGCTGCTA TCGAGATAGG ACGAGGAGGT TAGCCACGAG CGTATCTGAG ACTGGGAGAC GAACCACGCG TAGCAGCAGA GCACTAGCTC ---------- -------ATG GCCCGCGCTT
HvMpc1-H3-DOM    ACTGCACGGA CCTGCTGCTA TCGAGATAGG ACGAGGAGGT TAGCCACGAG CGTATCTGAG ACTGGGAGAC GAACCACGCG TAGCAGCAGA GCACTAGCTC ---------- -------ATG GCCCGCGCAT
HvMpc1-H3-REC    ACTGCACGGA CCTGCTGCTA TCGAGATAGG ACGAGGAGGT TAGCCACGAG CGTATCTGAG ACTGGGAGAC GAACCACGCG TAGCAGCAGA GCACTAGCTC CAGCAGAGCA CTAGCTCATG GCCCGCGCAT

                 131                                                                                                                        260
HvMpc1-H3-BW     TCCATGCAAC CGAGAGTCCA GGTCCTGCGC CCCAACACGC TACGCCTAGA GGAGTCGCCA CCGCTATCTT TCCAGACCAG GTGCGAGAGA TCGTAGGGGG CCTAACGAGC TGAAGTATTG GATCCGGGTA
HvMpc1-H3-BA     TCCATGCAAC CGAGAGTCCA GGTCCTGCGC CCCAACACGC TACGCCTGGA GGAGTCGCCA CCGCTATCTT TCCAGACCAG GTGCGAGAGA TCGTAGGGGG CCTAACGAGC TGAAGTATTG GATCCGGGTA
HvMpc1-H3-PLP    TCCATGCAAC CGAGAGTCCA GGTCCTGCGC CCCAACACGC TACGCCTAGA GGAGTCGCCA CCGCTATCTG TCCAGACCAG GTGGGAGAGA TCGTAGGGGG CCTAACGAGC TGAAGTATTG GATCCGGGTA
HvMpc1-H3-DOM    TCCATGCAAC CGAGAGTCCA GGTCCTGCGC CCCAACACGC TACGCCTGGA ATAGTCGCCA CCGCTATCTT TCCAGACCAG GTGCGAGAGA TCGTAGGGGA CCTAACGAGC TGAAGTATTG GATCCGGGTA
HvMpc1-H3-REC    TCCATGCAAC CGAGAGTCCA GGTCCTGCGC CCCAACACGC TACGCCTAGA GGAGTCGCCA CCGCTATCTT TCCAGACCAG GTGCGAGAGA TCGTAGGGGG CCTAACGAGC TGAAGTATTG GATCCGGGTA

                 261                                                                                                                        390
HvMpc1-H3-BW     TATAAGACTT GGACGGATAT CATCATTAGT GCAAGTGGAG ATGTATAGGC GGCGGAACGG CGGTGCTGGA CGTCCGTGTT CATTACACGG ATGGATCCTT GCTAGGCGTT TGGTGTACCT AATTCGGACT
HvMpc1-H3-BA     TATAAGACTT GGACGGATAT CATCATTAGT GCAAGTGGAG ATGTATAGGC GGCGGAACGG CGGTGCTGGA CGTCCGTGTT CATTACACGG ATGGATCCTT GCTAGGCGTT TGGTGTACCT AATTCGGACT
HvMpc1-H3-PLP    TATAAGACTT GGACGGATAT CATCATTAGT GCAAGTGGAG ATGTATAGGC GGCGGAACGG CGGTGCTGGA CGTCCGTGTT CATTACACGG ATGGATCCTT GCTAGGCGTT TGGTGTACCT AATTCGGACT
HvMpc1-H3-DOM    TATAAGACTT GGACGGATAT CATCATTAGT GCAAGTGGAG ATGTATAGGC GGCGGAACGG CGGTGCTGGA CGTCCGTGTT CATTACACGG ATGGATCCTT GCTAGGCGTT TGGTGTACCT AATTCGGACT
HvMpc1-H3-REC    TATAAGACTT GGACGGATAT CATCATTAGT GCAAGTGGAG ATGTATAGGC GGCGGAACGG CGGTGCTGGA CGTCCGTGTT CATTACACGG ATGGATCCTT GCTAGGCGTT TGGTGTACCT AATTCGGACT

                 391                                                                                                      494
HvMpc1-H3-BW     TAGGCGGTGC CCACATGGTG TGTTAATAAA GCACACCGGC AACCTGCCCA CGGACCCAAA CACAGCACAC ACAGGCAGTG GGGAAAAGAG ATGAGATGGG GAGG
HvMpc1-H3-BA     TAGGCGGTGC CCACATGGTG TGTTAATAAA GCACACCGGC AACCTGCCCA CGGACCCAAA CACAGCACAC ACAGGCAGTG GGGAAAAGAG ATGAGATGGG GAGG
HvMpc1-H3-PLP    TAGGCGGTGC CCACATGGTG TGTTAATAAA GCACACCGGC AACCTGCCCA CGGACCCAAA CACAGCACAC ACAGGCAGTG GGGAAAAGAG ATGAGATGGG GAGG
HvMpc1-H3-DOM    TAGGCGGTGC CCACATGGTG TGTTAATAAA GCACACCGGC AACCTGCCCA CGGACCCAAA CACAGCACAC ACAGGCAGTG GGGAAAAGAG ATGAGATGAG GAGG
HvMpc1-H3-REC    TAGGCGGTGC CCACATGGTG TGTTAATAAA GCACACCGGC AACCTGCCCA CGGACCCAAA CACAGCACAC ACAGGCAGTG GGGAAAAGAG ATGAGATGGG GAGG
```
