# Supplementary material for: Structural and functional divergence of the Mpc1 genes in wheat and barley
Source: BMC Evol Biol. 2019 Feb 26;19(Suppl 1):45. doi: 10.1186/s12862-019-1378-3 (PMC6391766; doi:10.1186/s12862-019-1378-3)
Supplement: Supplementary file 2 — Putative cis-acting regulatory elements identified in the Mpc1 promoters. Promoter analysis was performed using New PLACE database. “+” – coding strand, “–” – template strand. (PDF 326 kb) [file 12862_2019_1378_MOESM2_ESM.pdf]

Additional file 2. Putative cis-acting regulatory elements identified in the *Mpc1* promoters. Promoter analysis was performed using New PLACE database. "+" – coding strand, "–" – template strand.

>promoter_TaMpc1-A2

TTGAGAAAATATTAATATTAAAATAAAAAATACATTTGTATTTTATGATATTTTTAAAAAAACCAACTGAAAATCGTAAAATTAGAGAC
TTCGAGAAAAAAACAATTGACAACTGCAGGGAGCCGATGAAAGACAAATGGGCCGGCCCATATTGTGAGCGCTTGGACAACGATCCATA
GGACTTGTCATAGAAATTTGGCTGCATCCGAGCTGCCGGCGATGTATGACGGCGAAGAAACGGCCGCGCTGTGCATCCCGATTCATTGA
ACACATGGAGCTTTCCTAGCCTTTTGGTCCATCTACCTCTAGAGAGAAGTGTCGATCACGATGACCATGGCTGAGGGTGCTAAGCAGGT
CCACGTAGACGCGCTACTGTACAATGCTTAACAGGTCCACGTAGACGCTCTGCTGTACACTGCTCAACGGGGCCACGTAGACGCGCCTA
TGAATAAATACGGCTACAGCCCTGCTGCCCACAGATGACAGACCCCCCCCCCCCCCCCTCTCTCTCTCTCTCTCTAACACAATACACAC
CGGAGATGAGGTGAGATAGAGAGGTTAGTTCAGAGAAAGAGGAAGAGGAAGATAGAGAGGGAGAGA

Factor or Site Name	Loc.(Str.)	Signal Sequence	SITE #
GT1CONSENSUS	5 (+)	GRWAAW	S000198
MYCCONSENSUSAT	33 (-)	CANNTG	S000407
MYCCONSENSUSAT	33 (+)	CANNTG	S000407
GATABOX	47 (+)	GATA	S000039
MYB1AT	60 (+)	WAACCA	S000408
MYCCONSENSUSAT	64 (-)	CANNTG	S000407
MYCCONSENSUSAT	64 (+)	CANNTG	S000407
MYB2CONSENSUSAT	64 (+)	YAACKG	S000409
GT1CONSENSUS	69 (+)	GRWAAW	S000198
GT1CONSENSUS	95 (+)	GRWAAW	S000198
MYCCONSENSUSAT	103 (-)	CANNTG	S000407
CAATBOX1	103 (+)	CAAT	S000028
MYCCONSENSUSAT	103 (+)	CANNTG	S000407
CAATBOX1	105 (-)	CAAT	S000028
WRKY71OS	107 (+)	TGAC	S000447
MYCCONSENSUSAT	110 (-)	CANNTG	S000407
MYCCONSENSUSAT	110 (+)	CANNTG	S000407
MYB2CONSENSUSAT	110 (+)	YAACKG	S000409
DOFCOREZM	129 (+)	AAAG	S000265
MYCCONSENSUSAT	134 (-)	CANNTG	S000407
MYCCONSENSUSAT	134 (+)	CANNTG	S000407
SORLIP2AT	139 (+)	GGGCC	S000483
SORLIP2AT	144 (-)	GGGCC	S000483
CAATBOX1	151 (-)	CAAT	S000028
WRKY71OS	185 (-)	TGAC	S000447
WRKY71OS	225 (+)	TGAC	S000447
MYBCOREATCYCB1	237 (+)	AACGG	S000502
CGCGBOXAT	242 (-)	VCGCGB	S000501
CGCGBOXAT	242 (+)	VCGCGB	S000501
CAATBOX1	263 (-)	CAAT	S000028
MYCCONSENSUSAT	269 (-)	CANNTG	S000407
MYCCONSENSUSAT	269 (+)	CANNTG	S000407
DOFCOREZM	278 (-)	AAAG	S000265
DOFCOREZM	288 (-)	AAAG	S000265
CBFHV	318 (-)	RYCGAC	S000497
WRKY71OS	329 (+)	TGAC	S000447
CGCGBOXAT	365 (-)	VCGCGB	S000501
CGCGBOXAT	365 (+)	VCGCGB	S000501
CAATBOX1	378 (+)	CAAT	S000028
MYBCORE	385 (-)	CNGTTR	S000176
MYB2CONSENSUSAT	421 (+)	YAACKG	S000409
SORLIP2AT	426 (+)	GGGCC	S000483
BOXIIPCCHS	428 (-)	ACGTGGC	S000229
SORLIP1AT	428 (+)	GCCAC	S000482
CGCGBOXAT	437 (-)	VCGCGB	S000501
CGCGBOXAT	437 (+)	VCGCGB	S000501
MYCCONSENSUSAT	477 (-)	CANNTG	S000407
MYCCONSENSUSAT	477 (+)	CANNTG	S000407
WRKY71OS	481 (+)	TGAC	S000447
CAATBOX1	525 (+)	CAAT	S000028
GATABOX	549 (+)	GATA	S000039
MYB1LEPR	558 (+)	GTTAGTT	S000443
DOFCOREZM	570 (+)	AAAG	S000265

GATABOX	585 (+)	GATA	S000039

>promoter_TaMpc1-B2

GAGTTGACGGACGAAATCCACGCTTATCAATTTGTCGAGGGACCAAAAATAGACTTTTCTCATTAGTAAAAGCTCAACCTTCTCTCAGC
ATCTGGTGGGAAGGAACCTTGGCATGCCTCTGAGGCTCTGAGCACCCGCAACTTCCGGGGAGTCGACTGGCAGCATGTCCCTCTTCTAA
TAGTAAATGTTCGTGTTTCCCCCCACCTAGACATACAAATGCAACGATTCTTAGGACTTGTCACAGAAATATGACATCAATCAAACCTG
CCGGCGAAGAAACGGCCGCGCTGCGCATCCCGATTCATTGCACACATGGAGCTTTCCTTGCCTTTTGGTCCATCCACCTCTAGAGAGAA
GTGTCGATCACGATAACCATGGCTGAGGGTGCTAAGCAGGTCCACGTAGACGCGCTATTGTACAATGCTAAACAGGTCCACGTAGCCGC
GCTGCTGTACAATGCTGAACGGGGCCACGTAGACGCGCTGATGTATATAAATACGGCAACAATCCTGCCGCCCACAGATGAGATGACAT
AGAGAGAGGTTGCCTCAGAAGAAAGAGGAAGATAGAAAGAGAGAGAGAGAGAGAGAGAGAGAGA

Factor or Site Name	Loc.(Str.)	Signal Sequence	SITE #
WBOXATNPR1	4 (+)	TTGAC	S000390
WRKY71OS	5 (+)	TGAC	S000447
IBOX	23 (-)	GATAAG	S000124
GATABOX	25 (-)	GATA	S000039
CAATBOX1	28 (+)	CAAT	S000028
DOFCOREZM	54 (-)	AAAG	S000265
DOFCOREZM	69 (+)	AAAG	S000265
MYCCONSENSUSAT	89 (-)	CANNTG	S000407
MYCCONSENSUSAT	89 (+)	CANNTG	S000407
CBFHV	151 (-)	RYCGAC	S000497
CBFHV	151 (+)	RYCGAC	S000497
E2FCONSENSUS	194 (+)	WTTSSCSS	S000476
MYCCONSENSUSAT	214 (-)	CANNTG	S000407
MYCCONSENSUSAT	214 (+)	CANNTG	S000407
WRKY71OS	238 (-)	TGAC	S000447
WRKY71OS	250 (+)	TGAC	S000447
CAATBOX1	256 (+)	CAAT	S000028
MYBCOREATCYCB1	278 (+)	AACGG	S000502
CGCGBOXAT	283 (-)	VCGCGB	S000501
CGCGBOXAT	283 (+)	VCGCGB	S000501
CAATBOX1	304 (-)	CAAT	S000028
MYCCONSENSUSAT	310 (-)	CANNTG	S000407
MYCCONSENSUSAT	310 (+)	CANNTG	S000407
DOFCOREZM	319 (-)	AAAG	S000265
DOFCOREZM	329 (-)	AAAG	S000265
CBFHV	359 (-)	RYCGAC	S000497
GATABOX	368 (+)	GATA	S000039
IBOXCORE	368 (+)	GATAA	S000199
MYB1AT	370 (+)	WAACCA	S000408
CGCGBOXAT	406 (-)	VCGCGB	S000501
CGCGBOXAT	406 (+)	VCGCGB	S000501
CAATBOX1	413 (-)	CAAT	S000028
CAATBOX1	419 (+)	CAAT	S000028
CGCGBOXAT	442 (-)	VCGCGB	S000501
CGCGBOXAT	442 (+)	VCGCGB	S000501
CAATBOX1	455 (+)	CAAT	S000028
MYBCOREATCYCB1	463 (+)	AACGG	S000502
SORLIP2AT	467 (+)	GGGCC	S000483
BOXIIPCCHS	469 (-)	ACGTGGC	S000229
SORLIP1AT	469 (+)	GCCAC	S000482
CGCGBOXAT	478 (-)	VCGCGB	S000501
CGCGBOXAT	478 (+)	VCGCGB	S000501
CAATBOX1	505 (+)	CAAT	S000028
MYCCONSENSUSAT	520 (-)	CANNTG	S000407
MYCCONSENSUSAT	520 (+)	CANNTG	S000407
WRKY71OS	529 (+)	TGAC	S000447
DOFCOREZM	556 (+)	AAAG	S000265
GATABOX	565 (+)	GATA	S000039
DOFCOREZM	570 (+)	AAAG	S000265

>promoter_TaMpc1-D2

CCATCTGTGAAAAATGACGCGGCAATGCGTGCGGGAGCCGCTAGTAAAATTAANTGAGGAAAAACACTCCATCTGTGAAAAATGACGCG
GCAATGCGTGCGGGAGCCGCTAGTAACATTAATGACGGGATTAAATGCTGGTGTTTCCCTCCACCTGGACATAAAAACGCAACGATCCA
TAGGACTTGTCACAGAAATTTGGCTGCGTCAGGGCTGCCGGCGATATTATGACGGCGAAGAAACGGCCGCGCAGTACATCCCGATTTAT
TGCACACATGGAGCTTTCATAGCCTTTTGGCCCATCTACCTCTAGAGAGAAGTGTCGATCACGATGACCATGGGTTGAGGGTGCTAAGC
AGGTCCACGTAGACGCGCTACTGTACAATGCTTAATAGGTCCACGTAGACGCTCTCCTGTACAACGCTGAACAGGGCCACGTAGACCCG
CTGCTGAATAAGTACGGCAACAACCCTGCTGCCCACAGATGACAGACCCCCCTCTCACTCTCTCTCTCTCTCTAACACAACACATACT
GGAGATGAGATGAGATAAGAGAGGTTAGTTCAGAGAAAGAGGAAGAGGAAGATAGAGAGGGGAGAGA

**Factor or Site Name**	**Loc.(Str.)**	**Signal Sequence**	**SITE #**
MYCCONSENSUSAT	2 (-)	CANNTG	S000407
MYCCONSENSUSAT	2 (+)	CANNTG	S000407
GT1CONSENSUS	9 (+)	GRWAAW	S000198
WRKY71OS	15 (+)	TGAC	S000447
CGCGBOXAT	17 (-)	VCGCGB	S000501
CGCGBOXAT	17 (+)	VCGCGB	S000501
E2FCONSENSUS	19 (-)	WTTSSCSS	S000476
CAATBOX1	23 (+)	CAAT	S000028
GT1CONSENSUS	58 (+)	GRWAAW	S000198
GT1CONSENSUS	59 (+)	GRWAAW	S000198
MYCCONSENSUSAT	70 (-)	CANNTG	S000407
MYCCONSENSUSAT	70 (+)	CANNTG	S000407
GT1CONSENSUS	77 (+)	GRWAAW	S000198
WRKY71OS	83 (+)	TGAC	S000447
CGCGBOXAT	85 (-)	VCGCGB	S000501
CGCGBOXAT	85 (+)	VCGCGB	S000501
E2FCONSENSUS	87 (-)	WTTSSCSS	S000476
CAATBOX1	91 (+)	CAAT	S000028
WRKY71OS	122 (+)	TGAC	S000447
MYCCONSENSUSAT	151 (-)	CANNTG	S000407
MYCCONSENSUSAT	151 (+)	CANNTG	S000407
WRKY71OS	187 (-)	TGAC	S000447
WRKY71OS	206 (-)	TGAC	S000447
GATABOX	221 (+)	GATA	S000039
WRKY71OS	228 (+)	TGAC	S000447
MYBCOREATCYCB1	240 (+)	AACGG	S000502
CGCGBOXAT	245 (-)	VCGCGB	S000501
CGCGBOXAT	245 (+)	VCGCGB	S000501
CAATBOX1	266 (-)	CAAT	S000028
MYCCONSENSUSAT	272 (-)	CANNTG	S000407
MYCCONSENSUSAT	272 (+)	CANNTG	S000407
DOFCOREZM	281 (-)	AAAG	S000265
DOFCOREZM	291 (-)	AAAG	S000265
E2FCONSENSUS	293 (+)	WTTSSCSS	S000476
SORLIP2AT	296 (-)	GGGCC	S000483
CBFHV	321 (-)	RYCGAC	S000497
WRKY71OS	332 (+)	TGAC	S000447
WBOXNTERF3	332 (+)	TGACY	S000457
CGCGBOXAT	369 (-)	VCGCGB	S000501
CGCGBOXAT	369 (+)	VCGCGB	S000501
CAATBOX1	382 (+)	CAAT	S000028
SORLIP2AT	430 (+)	GGGCC	S000483
BOXIIPCCHS	432 (-)	ACGTGGC	S000229
SORLIP1AT	432 (+)	GCCAC	S000482
MYCCONSENSUSAT	481 (-)	CANNTG	S000407
MYCCONSENSUSAT	481 (+)	CANNTG	S000407
WRKY71OS	485 (+)	TGAC	S000447
SORLIP5AT	498 (-)	GAGTGAG	S000486
GATABOX	548 (+)	GATA	S000039
IBOX	548 (+)	GATAAG	S000124
MYB1LEPR	558 (+)	GTTAGTT	S000443
DOFCOREZM	570 (+)	AAAG	S000265
GATABOX	585 (+)	GATA	S000039

>promoter_TaMpc1-D3

TGGTTTAGCTTTAGCCCTAGGCTTCGAGAAATCCTGGCTCCGTCTCTGTTTTACACGTTCCTACTTTTCAACAATTTATAGATATTATG
ACCAAAGGCTTACCTACGGCGTCATTTGAGGAGTTCCGGTACAGTCTTTGCGTCAGCCGCGGTGCCGCTTCGACTGCGGGGGGTGTTGA
GTATATGTGTTATGTGTATATTGTGTATTGGGCCCGCCTCATAGTTCCTTATATAGTTGAGGTCCGTGACCCACCGTTGTACATCATAT
ATATGTGCCTATGCACGAGAGCAATACATCGTGCAATCATAGTCTCATACACTAGCGATCTGAAGCTTTACACCAGGTTATAGGTTTTC
TTCCTCGACTTTGGACGGATATCATCATCGGTGCCGCCAGTGAGATGTATAGGCGGCGGAACGGCGGTGCTGAACGTCCGTGTTCATTG
CACAGATGGATCCTTTGCTAGCCGTTTGGTGTACCTAACTCGGACTTAGGTGTTGCTTGCGGTGACCACATGGTGTATTAATAAAGCAC
AACGGCCTTCTGCCCACGGATCAAACACAGTACACACGGGCAGTTGGGAAAAAGATAACGGGGAGG

Factor or Site Name	Loc.(Str.)		Signal Sequence	SITE #
MYB1AT	1	(-)	WAACCA	S000408
DOFCOREZM	9	(-)	AAAG	S000265
DOFCOREZM	64	(-)	AAAG	S000265
CAATBOX1	72	(+)	CAAT	S000028
GATABOX	81	(+)	GATA	S000039
WRKY71OS	88	(+)	TGAC	S000447
WBOXNTERF3	88	(+)	TGACY	S000457
DOFCOREZM	93	(+)	AAAG	S000265
WRKY71OS	110	(-)	TGAC	S000447
MYCCONSENSUSAT	112	(-)	CANNTG	S000407
MYCCONSENSUSAT	112	(+)	CANNTG	S000407
DOFCOREZM	135	(-)	AAAG	S000265
WRKY71OS	141	(-)	TGAC	S000447
CGCGBOXAT	146	(-)	VCGCGB	S000501
CGCGBOXAT	146	(+)	VCGCGB	S000501
CAATBOX1	198	(-)	CAAT	S000028
CAATBOX1	205	(-)	CAAT	S000028
SORLIP2AT	208	(+)	GGGCC	S000483
SORLIP2AT	209	(-)	GGGCC	S000483
WRKY71OS	245	(+)	TGAC	S000447
WBOXNTERF3	245	(+)	TGACY	S000457
MYB2CONSENSUSAT	252	(-)	YAACKG	S000409
CAATBOX1	289	(+)	CAAT	S000028
CAATBOX1	301	(+)	CAAT	S000028
DOFCOREZM	333	(-)	AAAG	S000265
DOFCOREZM	365	(-)	AAAG	S000265
MYBST1	373	(+)	GGATA	S000180
GATABOX	374	(+)	GATA	S000039
GATABOX	376	(-)	GATA	S000039
MYBCOREATCYCB1	416	(+)	AACGG	S000502
CAATBOX1	442	(-)	CAAT	S000028
MYCCONSENSUSAT	448	(-)	CANNTG	S000407
MYCCONSENSUSAT	448	(+)	CANNTG	S000407
DOFCOREZM	458	(-)	AAAG	S000265
MYBCOREATCYCB1	467	(-)	AACGG	S000502
WRKY71OS	508	(+)	TGAC	S000447
WBOXNTERF3	508	(+)	TGACY	S000457
MYCCONSENSUSAT	512	(-)	CANNTG	S000407
MYCCONSENSUSAT	512	(+)	CANNTG	S000407
DOFCOREZM	528	(+)	AAAG	S000265
MYB2CONSENSUSAT	534	(+)	YAACKG	S000409
MYCCONSENSUSAT	575	(-)	CANNTG	S000407
MYB2CONSENSUSAT	575	(-)	YAACKG	S000409
MYCCONSENSUSAT	575	(+)	CANNTG	S000407
GT1CONSENSUS	581	(+)	GRWAAW	S000198
GT1CONSENSUS	582	(+)	GRWAAW	S000198
DOFCOREZM	585	(+)	AAAG	S000265
GATABOX	588	(+)	GATA	S000039
IBOXCORE	588	(+)	GATAA	S000199
MYB2CONSENSUSAT	590	(+)	YAACKG	S000409

>promoter_TaMpc1-D4

GGCTATGCCGTAGGCATACCTCTGCCATAGATATGCCGACGGCCGCGGTTACCTCCCGTCGGCGTAGTTTCAACGCCGTCAAACGGTCA
GCACTAACCGGGTAGGTTGGCCCGGTCTATGCCGACGACCGCGCCTATGCCGACGGTCTCCGTAGGCGTATTTCGAGCGGTCCCGACGG
CCTCGTCTGTGCCGACGGCCCCGTCGGCATAGATGGATGTATGCTGACGGCTTTTCTACGCCTACGGCCTGTCCTTGGCCGTCGGCATA
GGCCCATCTATGCCGACGGGGGCCGTCGGCATAGAGGAGGCCGTCGGCGCAAGCAGTTATTCTGGTAGTGAAAAACGGCCGCGCTGTTC
ATTGCACAGATGGAGTCTTCCTAGCCTTTTGATCCATCTAACTCTGAGATAAGTGTCGCTCACGATGGCCGTGGGTTGAGGGTGCAAAG
CATGTCCACGTAGACGCGCTACTGTACAATGATAACAGGTCCACGTTGACGCGCTGGTGAATAAATACGGCAACAACCCCGCTGCCCGC
AGATGACAGACCCTCGCTCACTCGCTCTCTAACACAACACCCACTGGAGAGAGAGAGAGAGAGAGA

Factor or Site Name	Loc.(Str.)	Signal Sequence	SITE #
GATABOX	30 (+)	GATA	S000039
DRECRTCOREAT	35 (+)	RCCGAC	S000418
CGCGBOXAT	43 (-)	VCGCGB	S000501
CGCGBOXAT	43 (+)	VCGCGB	S000501
MYBCORE	46 (+)	CNGTTR	S000176
DRECRTCOREAT	58 (-)	RCCGAC	S000418
WRKY71OS	78 (-)	TGAC	S000447
MYBCOREATCYCB1	82 (+)	AACGG	S000502
WRKY71OS	86 (-)	TGAC	S000447
MYBCORE	94 (-)	CNGTTR	S000176
MYBPZM	100 (-)	CCWACC	S000179
MYBPZM	104 (-)	CCWACC	S000179
SORLIP2AT	108 (-)	GGGCC	S000483
DRECRTCOREAT	120 (+)	RCCGAC	S000418
CGCGBOXAT	128 (-)	VCGCGB	S000501
CGCGBOXAT	128 (+)	VCGCGB	S000501
DRECRTCOREAT	138 (+)	RCCGAC	S000418
DRECRTCOREAT	189 (+)	RCCGAC	S000418
SORLIP2AT	195 (-)	GGGCC	S000483
DRECRTCOREAT	201 (-)	RCCGAC	S000418
WRKY71OS	223 (+)	TGAC	S000447
DOFCOREZM	229 (-)	AAAG	S000265
DRECRTCOREAT	259 (-)	RCCGAC	S000418
SORLIP2AT	268 (-)	GGGCC	S000483
DRECRTCOREAT	279 (+)	RCCGAC	S000418
SORLIP2AT	287 (+)	GGGCC	S000483
DRECRTCOREAT	292 (-)	RCCGAC	S000418
DRECRTCOREAT	310 (-)	RCCGAC	S000418
MYB2CONSENSUSAT	321 (-)	YAACKG	S000409
MYBCORE	321 (+)	CNGTTR	S000176
GT1CONSENSUS	337 (+)	GRWAAW	S000198
MYBCOREATCYCB1	341 (+)	AACGG	S000502
CGCGBOXAT	346 (-)	VCGCGB	S000501
CGCGBOXAT	346 (+)	VCGCGB	S000501
CAATBOX1	357 (-)	CAAT	S000028
MYCCONSENSUSAT	363 (-)	CANNTG	S000407
MYCCONSENSUSAT	363 (+)	CANNTG	S000407
DOFCOREZM	382 (-)	AAAG	S000265
GATABOX	404 (+)	GATA	S000039
IBOX	404 (+)	GATAAG	S000124
DOFCOREZM	442 (+)	AAAG	S000265
CGCGBOXAT	459 (-)	VCGCGB	S000501
CGCGBOXAT	459 (+)	VCGCGB	S000501
CAATBOX1	472 (+)	CAAT	S000028
GATABOX	476 (+)	GATA	S000039
IBOXCORE	476 (+)	GATAA	S000199
MYBCORE	478 (-)	CNGTTR	S000176
BP5OSWX	487 (-)	CAACGTG	S000436
WRKY71OS	492 (+)	TGAC	S000447
CGCGBOXAT	494 (-)	VCGCGB	S000501
CGCGBOXAT	494 (+)	VCGCGB	S000501
MYCCONSENSUSAT	534 (-)	CANNTG	S000407
MYCCONSENSUSAT	534 (+)	CANNTG	S000407
WRKY71OS	538 (+)	TGAC	S000447
SORLIP5AT	551 (-)	GAGTGAG	S000486

>promoter_HvMpc1-H2

ATGTTTGAATATAGTTACATGACACATAATTCCACAACGGGTGGGATGCTCCCTCCATGCTCACACACATCTGGGATGCCCCCCTTCGG
TTTTGGGGGGTCCGGGTGCCTCGTGCTGACACAGGTGAGCCGTCGAGCAAAAGCTACGTGCTCTGGCTTCTTTGGACTCAAAGGGCAAC
GATCCTTTTCAGTTGTCAAAAGAAATACGACATAGTACATCTCACCTGCCGGCGATCTTGGACGGCGAAGAAATGGCCGCGCTGTTCAT
TGGACAAATGGAGCCTTCCTAGCCTTTTGGTCCATCTAACTCTGAGATAAGTGTCGCTCACGATGACTGTGGATTGAAGGTGCAAAGCA
GGTCCACGTAGACGCGCTATTGTACAATGCTAAACGGGTCCACGTAGACGCGCTAATGACTAAATACGGCAACAACCCTGCTGCCCAAA
GATGACGGACCCTCACTCTCTCGCTCTCTAACACAACACAACACACACCGGAGAAGA

Factor or Site Name	Loc.(Str.)	Signal Sequence	SITE #
BIHD1OS	20 (-)	TGTCA	S000498
WRKY71OS	20 (+)	TGAC	S000447
MYB2CONSENSUSAT	35 (+)	YAACKG	S000409
MYCCONSENSUSAT	68 (-)	CANNTG	S000407
MYCCONSENSUSAT	68 (+)	CANNTG	S000407
BIHD1OS	116 (-)	TGTCA	S000498
WRKY71OS	116 (+)	TGAC	S000447
MYCCONSENSUSAT	121 (-)	CANNTG	S000407
MYCCONSENSUSAT	121 (+)	CANNTG	S000407
DOFCOREZM	139 (+)	AAAG	S000265
DOFCOREZM	159 (-)	AAAG	S000265
DOFCOREZM	169 (+)	AAAG	S000265
DOFCOREZM	183 (-)	AAAG	S000265
MYCCONSENSUSAT	188 (-)	CANNTG	S000407
MYB2CONSENSUSAT	188 (-)	YAACKG	S000409
MYCCONSENSUSAT	188 (+)	CANNTG	S000407
BIHD1OS	192 (+)	TGTCA	S000498
WRKY71OS	193 (-)	TGAC	S000447
DOFCOREZM	197 (+)	AAAG	S000265
MYCCONSENSUSAT	221 (-)	CANNTG	S000407
MYCCONSENSUSAT	221 (+)	CANNTG	S000407
CAATBOX1	266 (-)	CAAT	S000028
MYCCONSENSUSAT	272 (-)	CANNTG	S000407
MYCCONSENSUSAT	272 (+)	CANNTG	S000407
DOFCOREZM	291 (-)	AAAG	S000265
IBOX	313 (+)	GATAAG	S000124
WBOXHVISO1	331 (+)	TGACT	S000442
WRKY71OS	331 (+)	TGAC	S000447
WBOXNTERF3	331 (+)	TGACY	S000457
CAATBOX1	340 (-)	CAAT	S000028
DOFCOREZM	351 (+)	AAAG	S000265
OPAQUE2ZM22Z	359 (+)	TCCACGTAGA	S000017
CAATBOX1	375 (-)	CAAT	S000028
CAATBOX1	381 (+)	CAAT	S000028
MYBCOREATCYCB1	389 (+)	AACGG	S000502
OPAQUE2ZM22Z	395 (+)	TCCACGTAGA	S000017
WBOXHVISO1	413 (+)	TGACT	S000442
DOFCOREZM	443 (+)	AAAG	S000265
WRKY71OS	448 (+)	TGAC	S000447
SORLIP5AT	457 (-)	GAGTGAG	S000486

>promoter_HvMpc1-H3

CCTCTTTTCTACTGGTGCCGGCCAGTTCCGCGGCAGAGGACGCGACCGTTTAATTCGTTGCATGTCTACGATTCCTTTTTTTTTTATCG
TCAAATTAATTCGTCTCGCCTTCCTCTCGGGCGCCGACTGCACGGACCTGCTGCTATCGAGATAGGACGAGGAGGTTAGCCACGAGGGT
ATCTGAGACTGGGAGACGAACCACGCGTAGCAGCAGAGCACTAGCTCATGGCCCGCGCATTCCATGCAACCGAGAGTCCAGGTCCTGCG
CCCCAACACGCTACGCCTGGAGGAGTCGCCACCGCTATCTTTCCAGACCAGGTGCGAGAGATCGTAGGGGGCCTAACGAGCTGAAGTAT
TGGATCCGGGTATATAAGACTTGGACGGATATCATCATTAGTGCAAGTGGAGATGTATAGGCGGCGGAACGGCGGTGCTGGACGTCCGT
GTTCATTACACGGATGGATCCTTGCTAGGCGTTTGGTGTACCTAATTCGGACTTAGGCGGTGCCCACATGGTGTGTTAATAAAGCACAC
CGGCAACCTGCCCACGGACCCAAACACAGCACACACAGGCAGTGGGGAAAAGAGATGAGATGGGGAGG

Factor or Site Name	Loc.(Str.)	Signal Sequence	SITE #
DOFCOREZM	4 (-)	AAAG	S000265
CACTFTPPCA1	10 (+)	YACT	S000449
MYBCOREATCYCB1	46 (-)	AACGG	S000502
DOFCOREZM	75 (-)	AAAG	S000265
GT1CONSENSUS	83 (-)	GRWAAW	S000198
IBOXCORE	84 (-)	GATAA	S000199
GATABOX	85 (-)	GATA	S000039
WRKY71OS	89 (-)	TGAC	S000447
GATABOX	144 (-)	GATA	S000039
GATABOX	150 (+)	GATA	S000039
SORLIP1AT	168 (+)	GCCAC	S000482
GATABOX	178 (-)	GATA	S000039
CACTFTPPCA1	217 (+)	YACT	S000449
SORLIP2AT	228 (-)	GGGCC	S000483
MYBCORE	245 (-)	CNGTTR	S000176
SORLIP1AT	295 (+)	GCCAC	S000482
GATABOX	303 (-)	GATA	S000039
DOFCOREZM	306 (-)	AAAG	S000265
MYCCONSENSUSAT	316 (-)	CANNTG	S000407
MYCCONSENSUSAT	316 (+)	CANNTG	S000407
SORLIP2AT	336 (+)	GGGCC	S000483
CACTFTPPCA1	352 (-)	YACT	S000449
CAATBOX1	355 (-)	CAAT	S000028
MYBST1	383 (+)	GGATA	S000180
GATABOX	384 (+)	GATA	S000039
GATABOX	386 (-)	GATA	S000039
CACTFTPPCA1	396 (-)	YACT	S000449
MYCCONSENSUSAT	400 (-)	CANNTG	S000407
MYCCONSENSUSAT	400 (+)	CANNTG	S000407
CACTFTPPCA1	402 (-)	YACT	S000449
MYBCOREATCYCB1	424 (+)	AACGG	S000502
MYBPLANT	476 (-)	MACCWAMC	S000167
MYCCONSENSUSAT	510 (-)	CANNTG	S000407
MYCCONSENSUSAT	510 (+)	CANNTG	S000407
DOFCOREZM	526 (+)	AAAG	S000265
CACTFTPPCA1	575 (-)	YACT	S000449
GT1CONSENSUS	580 (+)	GRWAAW	S000198
DOFCOREZM	583 (+)	AAAG	S000265
